# Comparing The Effects of Small Molecules BIX-01294, Bay K8644, RG-108 and Valproic Acid, and Their Different Combinations on Induction of Pluripotency Marker-Genes by Oct4 in The Mouse Brain

**DOI:** 10.22074/cellj.2015.488

**Published:** 2015-01-13

**Authors:** Sareh Asadi, Samaneh Dehghan, Maryam Hajikaram, Seyed Javad Mowla, Abolhassan Ahmadiani Ahmadiani, Mohammad Javan

**Affiliations:** 1 Department of Physiology, Faculty of Medical Sciences, Tarbiat Modares University, Tehran, Iran; 2 Department of Stem Cells and Developmental Biology at Cell Science Research Center, Royan Institute for Stem Cell Biology and Technology, ACECR, Tehran, Iran; 3 Department of Molecular Genetics, Faculty of Basic Sciences, Tarbiat Modares University, Tehran, Iran; 4 Neuroscience Research Center, Shahid Beheshti Medical University, Tehran, Iran

**Keywords:** Oct4, Valproic Acid, Reprogramming, Pluripotency, Small Molecules

## Abstract

**Objective:**

Every cell type is characterized by a specific transcriptional profile together with a unique epigenetic landscape. Reprogramming factors such as Oct4, Klf4, Sox2 and c-Myc enable somatic cells to change their transcriptional profile and convert them to pluripotent cells. Small molecules such as BIX-01294, Bay K8644, RG-108 and valproic acid (VPA) are reported as effective molecules for enhancing induction of pluripotency *in vitro*, however, their effects during *in vivo* reprogramming are addressed in this experimental study.

**Materials and Methods:**

In this experimental study, Oct4 expressing lentiviral particles and small molecules BIX-01294, Bay K8644 and RG-108 were injected into the right ventricle of mice brain and VPA was systematically administered as oral gavages. Animals treated with different combinations of small molecules for 7 or 14 days in concomitant with Oct4 exogenous expression were compared for expression of pluripotency markers. Total RNA was isolated from the rims of the injected ventricle and quantitative polymerase chain reaction (PCR) was performed to evaluate the expression of endogenous Oct4, Nanog, c-Myc, klf4 and Sox2 as pluripotency markers, and Pax6 and Sox1 as neural stem cell (NSC) markers.

**Results:**

Results showed that Oct4 exogenous expression for 7 days induced pluripoten- cy slightly as it was detected by significant enhancement in expression of Nanog (p<0.05). Combinatorial administration of Oct4 expressing vector and BIX-01294, Bay K8644 and RG-108 did not affect the expression of pluripotency and NSC markers, but VPA treatment along with Oct4 exogenous expression induced Nanog, Klf4 and c-Myc (p<0.001). VPA treatment before the induction of exogenous Oct4 was more effective and significantly increased the expression of endogenous Oct4, Nanog, Klf4, c-Myc (p<0.01), Pax6 and Sox1 (p<0.001).

**Conclusion:**

These results suggest VPA as the best enhancer of pluripotency among the chemicals tested, especially when applied prior to pluripotency induction by Oct4.

## Introduction

In early embryos, cells undergo cascades of irreversible cell fate decisions. These fates are fixed by the presence of specific epigenetic landscape over the genome and cells thus become differentiated (1). Because of the limitation in the response of endogenous adult stem cells, especially in the central nervous system (CNS), endogenous repair cannot completely replace damaged cells with new ones (2). To circumvent limited regenerative capacity of the mammalian CNS, stem cellbased therapies are particularly attractive (3). The ground breaking work of Takahashi and Yamanaka (4) introduced an elegant way of restoring pluripotency in somatic cells. They used transcription factors that maintain pluripotency in embryonic stem (ES) cells to reprogram somatic cells and convert them to induced pluripotent stem cells (iPSCs). Oct4, Sox2, Klf4 and c-Myc are key transcription factors that facilitate differentiated cell reprogramming, albeit with a low efficiency. iPSCs are good candidates for stem cell therapy allowing the generation of patient-specific pluripotent cells and personalized disease modeling (5). However, there are several issues that need to be resolved before the application of the reprogramming strategy in regenerative medicine. Genomic alterations resulted from virus-mediated delivery of reprogramming factors and oncogenicity of Klf4 and c-Myc pose serious clinical concerns (6). Attempts have been made to reduce the number of transcription factors and substitute them with chemicals (7-9). Small molecules offer several advantages such as rapid, reversible and dose-dependent effects, structural diversity provided by synthetic chemistry and relative ease of handling and administration compared with genetic interventions (10). Moreover, by erasure of epigenetic marks (DNA methylation and histone modifications), they facilitate the alteration of transcriptional pattern of the cell. These molecules can increase reprogramming efficiency and push partial reprogrammed cells to the fully pl uripotent state (11).

Recent studies have introduced chemicals which are able to either enhance reprogramming efficiency or substitute some reprogramming factors. Valproic acid (VPA) is a histone deacetylase (HDAC) inhibitor which relieves HDAC-dependent transcriptional repression and causes hyperacetylation of histones in cultured cells and *in vivo* (12). VPA treatment for a week improved the percentage of Oct4-GFP-positive cells by more than 100-fold and 50-fold for three-factor (Oct4, Sox2 and Klf4) and four-factor (Oct4, Sox2, Klf4 and c-Myc) reprogramming respectively. VPA enhances reprogramming efficiency and consequently provides a chance for reducing the number of required reprogramming factors. In the presence of VPA, the three-factor-infected primary human fibroblasts could be reprogrammed at a rate which is 10- to 20-fold higher than previously reported efficiencies (9). Melton and colleagues demonstrated that in the presence of VPA, two factors (Oct4 and Sox2) were able to reprogram human fibroblasts and the efficiency was similar to that of the three-factors (13).

BIX-01294 is another small molecule which enables reprogramming of mouse embryonic fibroblasts (MEFs) into iPSCs in the absence of Sox2 expression and by only two exogenous factors Oct4 and Klf4 (7). A subsequent chemical screen in fibroblasts with BIX-01294, RG-108 (an identified DNA methyltransferase inhibitor) and Bay K8644 (an L-type calcium channel agonist that can work synergistically with BIX-01294) increased reprogramming efficiency (7, 8).

Although the chemical approach seems to be useful in combination with genetic strategies, effects of small molecules during *in vivo* reprogramming/trans differentiation need to be comparatively clarified. Most of them may have more than one target and unexpected toxicity or other *in vivo* side-effects may thus interfere with their clinical application (10). To study the effects of VPA, BIX-01294, RG-108 and Bay K8644 on inducing the expression of pluripotency markers *in vivo*, we used them in different combinations with lentiviruses containing Oct4 expression vectors. As reported, neural stem cells endogenously express Sox2 and Klf4 (14), therefore they can be reprogrammed just by one factor (Oct4) *in vitro* (15). In this study, we targeted the subventricular zone which contains NSCs and other neural cells to find the most effective combination of these chemicals for inducing pluripotency and neural stem cell (NSC) markers *in vivo*.

## Materials and Methods

### Animals

This experimental study was performed on C57BL/6 mice (Pasteur Institute, Tehran, Iran). The animals were 8 to 9 weeks of age (20-25 g) and were maintained in a temperature-controlled room under a 12 hour light/dark schedule. Water and food were available ad libitum. All practices were in accordance with NIH guidelines for the use of animals in research and approved by The Committee for Ethics in Research, Tarbiat Modares University. Efforts were made to minimize the suffering of animals and to reduce the number of animals used.

### Small molecules and Oct4 expressing virus

BIX-01294, (R)-(+)-Bay K8644 and RG-108 were purchased from Sigma-Aldrich, (Germany). VPA was used as sodium valproate (Abidi Co., Iran). Doxinducible Fuw-based lentiviral vectors that encoded mouse Oct4 cDNA (Royan Institute, Iran) were transfected by a Virapower Lentiviral Packaging Mix (Invitrogen, Carlsbad, CA) into 293T cells by using the Lipofectamine 2000 Transfection Reagent (Invitrogen, Carlsbad, CA). At 48 hours post-infection, viral supernatants were collected, filtered, concentrated and re-suspended. The final concentration of viral particles was approximately 700,000/ml. In the intracerebroventricular (i.c.v.) injections, the final volume of injections was adjusted to 2 μl and included the viral particles and chemical compounds.

### Animal stereotaxic surgery and intervention

In order to administer i.c.v. injections daily, we stereotaxically implanted a cannula just over the right cerebral ventricle in each animal (A: 3.6 and L: 1.1 from the lambda, V: 2.2 from the dura). To induce exogenous Oct4 expression, animals received 3 μl of medium containing viral particles followed by a 2 μl injection of doxycycline solution (6 ng/mouse, i.c.v.) over 7 consecutive days after the Oct4 injection. BIX-01294, Bay K8644 and RG-108 were injected through implanted cannula into the right brain ventricle for 7 days and the final volume for each injection was adjusted to 2 μl. Considering the brain water volume, the concentration of solutions was adjusted to achieve an i.c.v concentration about 1, 2 and 0.04 μM respectively. Animals received 150 mg/kg of VPA (as sodium valproate) twice daily via oral gavages for 7 days. All i.c.v. injections were administered into the right ventricle of each animal over a period of 10 minutes. Animal groups and the combination of reagents they received are summarized in Table 1. Two groups have VPA pretreatment for 7 days before phosphate buffered saline (PBS) and doxycycline (V+Vehicle & Dox) or Oct4 (V+O) i.c.v injections. Animals were decapitated at day 8 following viral or PBS administration.

**Table 1:** Animal interventions in different experimental groups


Group name	Interventions	Treatment period after Oct4 (or vehicle) injection (days)	Pre-treatment

**Intact**	-	-	-
**Vehicle**	PBS	7	-
**O7**	Oct4	7	-
**O14**	Oct4	14	-
**OBiBa**	Oct4+BIX+Bay K	7	-
**OBiR**	Oct4+BIX+RG	7	-
**OV**	Oct4+VPA	7	-
**OBiBaV**	Oct4+BIX+Bay K+VPA	7	-
**OBiRV**	Oct4+BIX+RG+VPA	7	-
**OBiBaRV**	Oct4+BIX+Bay K+RG+VPA	7	-
**V+Vehicle&Dox**	VPA+PBS+Dox	7	√
**V+O**	VPA+Oct4	7	√


BIX; BIX-01294, Bay K; Bay K8644, RG; RG-108, VPA; Valproic acid, Oct4; Oct4 expressing lentiviral particles, PBS; Phos-phate buffered saline and Dox; Doxycycline.

### RNA extraction and cDNA synthesis

The rims of injected ventricles (1 mm thick) from injected (right) ventricle were extracted and total RNA was isolated using a High Pure RNA Tissue Kit (Roche, Germany) according to the manufacturer’s instructions. Samples were used immediately for the reverse transcription reaction using oligo-dT primers (GeneOn, Germany) and RevertAid^TM^ Reverse Transcriptase (Fermentas, GMBH, Germany) based on the manufacturer’s protocol. The reactions were incubated at 42˚C for 60 minutes and then inactivated at 70˚C for 10 minutes. Produced cDNA was used in real-time PCR analysis to study the expression of pluripotency and NSC markers.

### Gene expression analysis

The cDNA pool was subjected to quantitative real-time PCR (q-PCR) by using a Real Q-PCR Master Mix Kit (Ampliqon, Herlev, Denmark) on a Rotor-Gene Q device (Qiagene, Hilden, Germany). The following conditions were used for q-PCR: initial heating for 15 minutes at 95˚C, 35 cycles of amplification, each composed of 60 seconds at 95˚C, 60 seconds at the annealing temperature and 60 seconds at 72˚C. The annealing temperature for glyceraldehyde 3-phosphate dehydrogenase (GAPDH), Sox2, total Oct4, Pax6 and Sox1 was 63˚C; for c-Myc, Nanog, endogenous Oct4 and Klf4 was 59˚C. Reactions were performed in duplicate. GAPDH was used as an endogenous control to minimize the effect of sample variations in calculating the relative expression levels of target genes by the delta delta Ct method. Primer sequences used for amplification were as follows: tOct4 (NM_013633.2) forward: 5ˊ GGAAAGCAACTCAGAGGGAAC 3ˊ; tOct4 reverse: 5ˊ AGCGACAGATGGTGGTCTG 3ˊ; eOct4 (NM_013633.2) forward: 5ˊ TCTTTCCACCAGGCCCCCGGCTC 3ˊ; eOct4 reverse: 5ˊ AGCGACAGATGGTGGTCTG 3ˊ; Nanog (NM_028016.2) forward: 5ˊ CCTCCAGCAGATGCAAGAA 3ˊ; Nanog reverse: 5ˊ GTGCTGAGCCCTTCTGAATC 3ˊ; Klf4 (NM_010637.3) forward: 5ˊ GGCGAGAAACCTTACCACTG 3ˊ; Klf4 reverse: 5ˊ TACTGAACTCTCTCTCCTGGC 3ˊ; c-Myc (NM_010849.4) forward: 5ˊ TCAAGCAGACGAGCACAAGC 3ˊ; c-Myc reverse: 5ˊ TACAGTCCCAAAGCCCCAGC 3ˊ; Sox2 (NM_011443.3) forward: 5ˊ GGTTACCTCTTCCTCCCACTCCAG 3ˊ; Sox2 reverse: 5ˊ TCACATGTGCGACAGGGGCAG 3ˊ; Pax6 (NM_013627.4) forward: 5ˊ AGTGAATGGGCGGAGTTATG 3ˊ; Pax6 reverse: 5ˊ ACTTGGACGGGAACTGACAC 3ˊ; Sox1 (NM_009233.3) forward: 5ˊ ATTTAATGGCAGCCCGGGCCCG 3ˊ; Sox1 reverse: 5ˊ GCGAGCAGAGAGCCAGAGAGCT 3ˊ; GAPDH (NM_008084.2) forward: 5ˊ GTGTTCCTACCCCCAATGTGT 3ˊ; GAPDH reverse: 5ˊ ATTGTCATACCAGGAAATGAGCTT 3ˊ.

The total Oct4 primers were designed to amplify an inner part of Oct4 cDNA which is common between the coding sequence of cloned Oct4 and the endogenous Oct4; the endogenous Oct4 primers were designed against the initial non-coding part of Oct4 mRNA and were able to amplify only the endogenous gene.

### Statistical analysis

Gene expression analysis was performed using one-way analysis of variance, followed by the Tukey post-test using GraphPad Prism 4.0 (Graph- Pad Software, San Diego, CA). P<0.05 was considered statistically significant.

## Results

To study the effects of small molecules on the efficacy of Oct4 for inducing pluripotency markers *in vivo*, we injected viral particles containing an inducible construct for Oct4.

**Fig 1:**
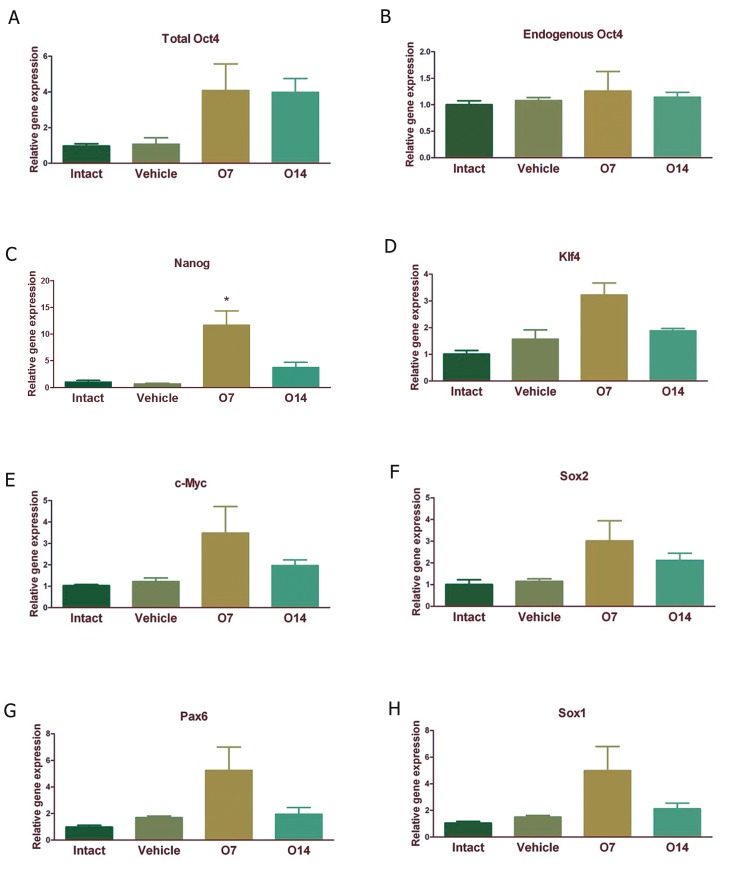
Quantitative analysis of pluripotency and neural stem cell (NSC) marker expression following forced expression of exogenous Oct4 in the tissue collected from the rims of injected brain ventricles. A. Total (Endogenous + exogenous) Oct4 expression, B. Endogenous Oct4 expression, C. Nanog expression, D. Klf4 expression, E. c-Myc expression, F. Sox2 expression, G. Pax6 expression and H. Sox1 expression in different groups. Results were normalized by GAPDH as a housekeeping gene. Vehicle; 7 days PBS i.c.v injection, O7; Oct4 expressing lentivirus i.c.v injection followed by doxycycline i.c.v injections for another 7 consecutive days and O14; Oct4 expressing lentivirus i.c.v injection followed by doxycycline i.c.v injections for another 14 consecutive days. Each group contains 5-7 animals. *; P<0.05.

As shown in figure 1A, 7 or 14 days injection of doxcycycline (i.c.v.) caused increased expression of total Oct4 within the rims of the injected ventricle, while the expression of endogenous Oct4 (eOct4) did not show a significant elevation (Fig 1B). The expression of pluripotency markers including Nanog (Fig 1C), Klf4 (Fig 1D), c-Myc (Fig 1E) and Sox2 (Fig 1F) were evaluated following the induction of exogenous Oct4. A more prominent increase in the expression of markers was observed on day 7 post-induction, but only the increase in the expression of Nanog was statistically significant. We also checked the expression of NSC markers following exogenous Oct4 induction. Again we observed a trend for the increased expression of NSC markers Pax6 (Fig 1G) and Sox1 (Fig 1H), especially on day 7 post-induction.

**Fig 2:**
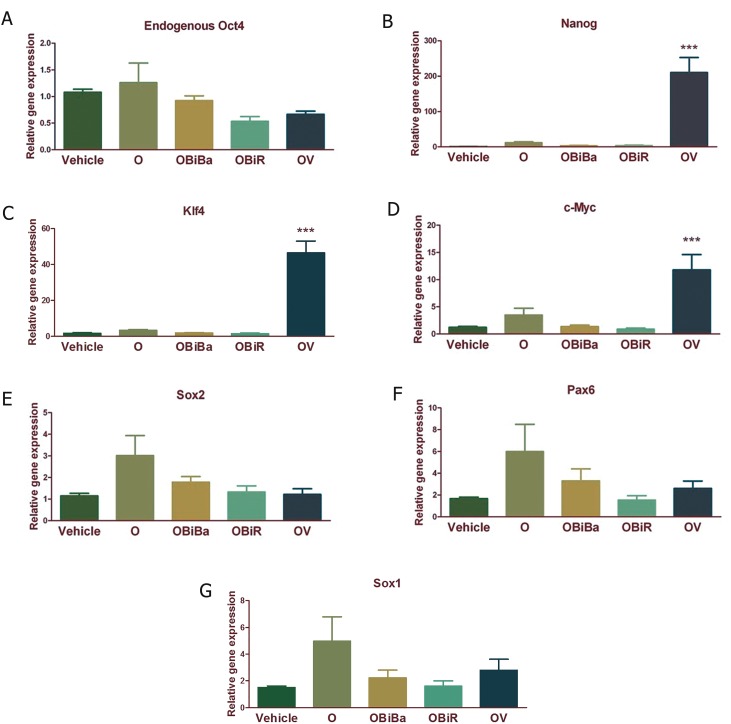
Quantitative analysis of pluripotency and neural stem cell (NSC) marker expression following exogenous Oct4 expression along with different small molecules including BIX-01294, Bay K8644, RG-108 and VPA in the tissue samples collected from the rims of injected brain ventricles. A. Endogenous Oct4 expression, B. Nanog expression, C. Klf4 expression, D. c-Myc expression, E. Sox2 expression, F. Pax6 expression and G. Sox1 expression in different groups. Results were normalized by GAPDH as a housekeeping gene. Vehicle; 7 days PBS i.c.v injection, O; Oct4 expressing lentivirus i.c.v injection followed by doxycycline i.c.v injections for another 7 consecutive days, OBiBa; Oct4 expressing lentivirus i.c.v injection followed by BIX, Bay K and doxycycline i.c.v injections for another 7 consecutive days, OBiR; Oct4 expressing lentivirus i.c.v injection followed by BIX, RG and doxycycline i.c.v injections for another 6 consecutive days and OV; Oct4 expressing lentivirus i.c.v injection followed by doxycycline i.c.v injections and VPA oral gavages for another 6 consecutive days. Each group contains 5-7 animals. ***; P<0.001.

The effect of *in vivo* expression of exogenous Oct4 on pluripotency and NSC markers was more remarkable on day 7 post-induction. Therefore, in the subsequent experiment, for checking the possible supportive effects of small molecules we undertook 7 day induction of Oct4 in the presence of different combinations of small molecules. We checked the effects of BIX-01294 +Bay K8644 in presence of Oct4 (OBiBa), BIX-01294 + RG-108 in presence of Oct4 (OBiR) and VPA in presence of Oct4 (OV) on the expression of both pluripotency and NSC markers (Fig 2A). Significant effects were only detected in the OV group for Nanog (Fig 2B), Klf4 (Fig 2C) and c-Myc (Fig 2D) (p<0.001). Non-significant changes were observed for eOct4 (Fig 2A), Sox2 (Fig 2E), Pax6 (Fig 2F) and Sox1 (Fig 2G).

**Fig 3:**
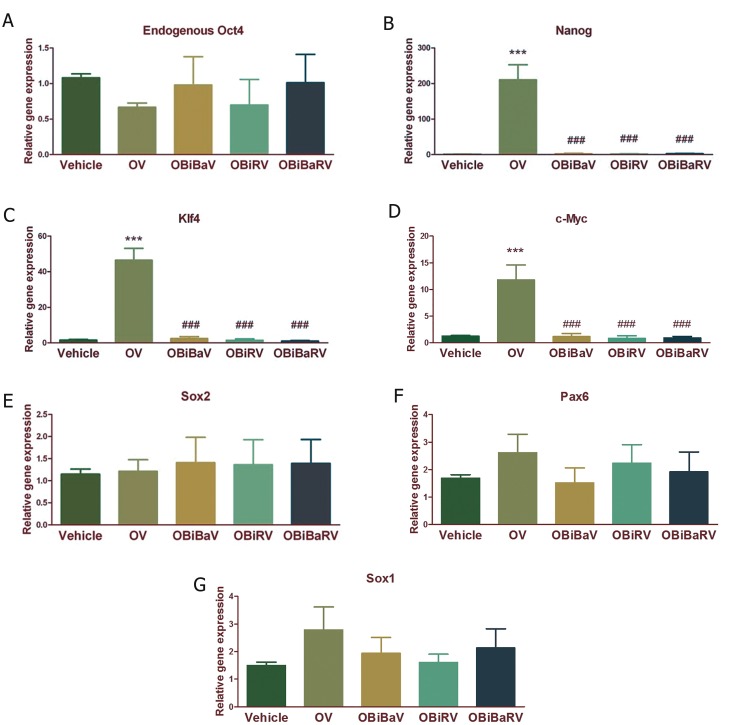
Quantitative analysis of pluripotency and neural stem cell (NSC) marker expression following exogenous Oct4 expression and VPA along with different small molecules including of BIX-01294, Bay K8644, RG-108 in the tissue collected from the rims of injected brain ventricles. A. Endogenous Oct4 expression, B. Nanog expression, C. Klf4 expression, D. c-Myc expression, E. Sox2 expression, F. Pax6 expression and G. Sox1 expression in different groups. Results were normalized by GAPDH as a housekeeping gene. Vehicle; 7 days PBS i.c.v injection, OV; Oct4 expressing lentivirus i.c.v injection followed by doxycycline i.c.v injections and VPA oral gavages for another 7 consecutive days, OBiBaV; Oct4 expressing lentivirus i.c.v injection followed by BIX, Bay k and doxycycline i.c.v injections and VPA oral gavages for another 7 consecutive days, OBiRV; Oct4 expressing lentivirus i.c.v injection followed by BIX, RG and doxycycline i.c.v injections and VPA oral gavages for another 7 consecutive days and OBiBaRV; Oct4 expressing lentivirus i.c.v injection followed by BIX, Bay k, RG and doxycycline i.c.v injections and VPA oral gavages for another 6 consecutive days. Each group contains 5-7 animals. ***; P<0.001 compared to Vehicle group and ###; P<0.001 compared to OV group.

Considering the significant effects of VPA on the induction of pluripotency markers by Oct4, we studied the possible supportive effects of BIX-01294 +Bay K8644, BIX-01294 + RG-108 and BIX-01294 +Bay K8644+ RG-108 on pluripotency induction by Oct4 and VPA. The groups were named OBiBaV, OBiRV, and OBiBaRV respectively (Fig 3). Not only these three mentioned combinations did not increase the expression of pluripotency and NSC markers (Fig 3 A-G), the expression of Nanog, Klf4 and c-Myc was significantly decreased (Fig 3 B-D, all p<0.001).

**Fig 4:**
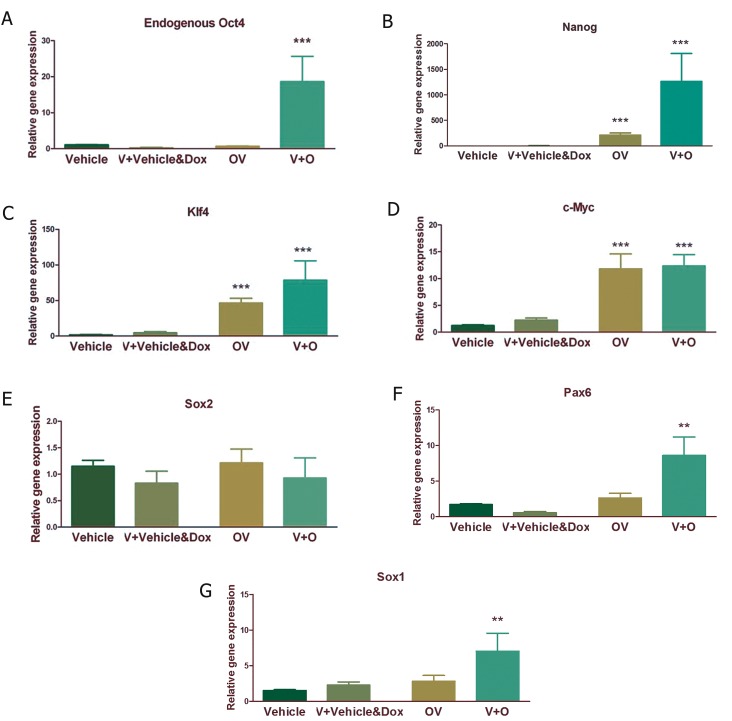
Quantitative analysis of pluripotency and neural stem cell (NSC) markers following exogenous Oct4 expression along with or after VPA treatment. A. Endogenous Oct4 expression, B. Nanog expression, C. Klf4 expression, D. c-Myc expression, E. Sox2 expression, F. Pax6 expression and G. Sox1 expression in different groups. Results were normalized by GAPDH as a housekeeping gene. Vehicle; 7 days PBS i.c.v injection, V+Vehicle & Dox; VPA oral gavages for 7 days prior to PBS and doxycycline i.c.v injection for another 7 days, OV; Oct4 expressing lentivirusi.c.v injection followed by doxycycline i.c.v injections and VPA oral gavages for another 7 consecutive days and V+O; VPA oral gavages for 7 days prior to Oct4 expressing lentivirus i.c.v injection followed by doxycycline i.c.v injections for another 7 consecutive days. Each group contains 5-7 animals. **; P<0.01 and ***; P<0.001.

Between the different small molecules and their different combinations which were administered in this study, only VPA was able to potentiate the effect of Oct4 on endogenous expression of pluripotency markers. For further characterization of the effects of VPA, we compared the effect of 7 day VPA administration as pretreatment and in conjunction with Oct4 induction (Fig 4). Seven day administration of VPA and the vehicle of viral particles and doxycycline (V+Vehicle & Dox) did not exert a remarkable effect on the expression of pluripotency and NSC markers. As mentioned earlier, the administration of VPA in conjunction with Oct4 (OV) caused significant increase in the expression of pluripotency markers Nanog, Klf4 and c-Myc. Interestingly, 7 day pretreatment with VPA followed by 7 days induction of Oct4 (V+O) caused significant increase in the expression of both pluripotency and NSC markers. It increased the expression of eOct4 (Fig 4A, p<0.001), Nanog (Fig 4B, p<0.001), Klf4 (Fig 4C, p<0.001), c-Myc (Fig 4D, p<0.001), Pax6 (Fig 4F, p<0.01) and Sox1 (Fig 4G, p<0.01).

## Discussion

Small molecules which can modulate specific target(s) in signaling and epigenetic mechanisms have been shown to be useful chemicals for manipulating cell fate (7-9). These chemicals enhance reprogramming of differentiated cells to pluripotent stem cells *in vitro* and provide the opportunity to minimize genetic manipulations of starting cells (16). Most of the small molecules have several targets; therefore, more extensive studies are required to fully understand their potential effects for clinical application. To our knowledge, there is no report concerning their effects on cellular transdifferentiation and reprogramming *in vivo*.

While exogenous Oct4 expression for 7 days increased only the expression of Nanog, its combined administration with VPA increased the induction of endogenous markers of pluripotency. Other small molecules and their combination with VPA did not increase the expression of pluripotency markers. When VPA was applied as a pretreatment, its effects were potentiated and in conjunction with Oct4 induced both pluripotency and NSC markers *in vivo*.

In a same but *in vitro* study, Medvedev et al. (17) derived human iPS cells from fetal neural stem cells by transfection with a polycistronic plasmid vector carrying the mouse Oct4, Sox2, Klf4, and c- Myc genes or a plasmid that expresses the human OCT4. They also showed that fetal stem cells can be more effectively reprogrammed by using VPA and BIX-01294.

Among Yamanak factors, c-Myc and Klf4 are oncogenic factors and their overexpression may lead to tumor development (18). Therefore omitting them from the reprogramming cocktail is an important achievement especially in *in vivo* studies. Sox2 was another pluripotency marker that we omitted in this study. Some neural cells within the brain especially the neural stem cells express Sox2. This may explain why the omission of Sox2 from Yamanaka factors in the current study did not interrupt the possibility of partial reprogramming.

Results of RT-PCR analysis provided evidence that Oct4 alone was sufficient for the induction of pluripotency markers *in vivo* when applied with VPA. VPA facilitates this process by inducing epigenetic instability within the starting cells by hyperacetylation of histones which leads to genomic instability (19). Interestingly, VPA administration before inducing Oct4 expression by doxcycycline was more effective. Expression levels of eOct4, Nanog and Klf4 (key ESC transcription factors) were higher in VPA pretreated animals which consequently received Oct4 induction (V+O) when compared with animals which received these two factors simultaneously (OV). Moreover, the expression of Pax6 and Sox1 as NSC markers was increased significantly only in the V+O animal group. This shows that when the epigenetic state is prepared before the induction of exogenous Oct4 expression, Oct4 transcription factor acts more effectively to induce reprogramming.

The only factor which remained unchanged during animal treatment in different groups was Sox2. Even in animals which received V+O, Sox2 expression level did not increase. NSCs endogenously express Sox2, so small changes in its expression level may not be detectable.

As it can be seen in figures 2 and 3, other small molecules (except VPA) were not able to potentiate the effect of exogenous Oct4 on the expression of pluripotency and NSC markers. Unexpectedly, they even reduced the expression of pluripotency markers to levels lower than those which were induced by Oct4+VPA. Overall, further work is required to examine the probable toxic effects of these molecules on neural cells, especially on those which are reprogrammed to pluripotent or neural stem cells.

## Conclusion

We show that Oct4 alone has the ability to induce expression of pluripotent markers *in vivo* especially in the presence of VPA. VPA was more effective when administrated as a pretreatment. Other small molecules including BIX-01294, Bay K8644 and RG-108 in different combinations did not exert any effect on Oct4 induced pluripotency.
